# Testing a Family Conflict Intervention for Parents and Typically Developing Adolescent Siblings of Individuals with Intellectual and/or Developmental Disabilities

**DOI:** 10.3390/ijerph21121666

**Published:** 2024-12-13

**Authors:** Vevette J. H. Yang, Kathleen N. Bergman, E. Mark Cummings

**Affiliations:** Department of Psychology, University of Notre Dame, Notre Dame, IN 46556, USA; kbergman@nd.edu (K.N.B.); ecumming@nd.edu (E.M.C.)

**Keywords:** family conflict, marital conflict, typically developing adolescents, intellectual and/or developmental disabilities, family intervention, conflict intervention, conflict discussion, adolescent adjustment

## Abstract

Parents and typically developing (TD) youth siblings of individuals with intellectual and/or developmental disabilities (IDD) often experience greater caregiving burden, stress, and hardships in family functioning. They are at increased risk of family conflict and youth adjustment problems when TD siblings are adolescents since they need to balance caregiving responsibilities and various changes that naturally occur during adolescence. However, there is a lack of intervention research on parents and TD adolescent siblings that focuses on family conflict and family-wide participation. This study analyzed whether participating in a brief family intervention could improve families’ knowledge of marital and family-wide conflict and TD adolescents’ adjustment problems. We found that mothers and fathers significantly improved their knowledge of marital conflict and that TD adolescents significantly improved their knowledge of family-wide conflict. We also found that fathers reported significant reductions in internalizing and externalizing problems in TD adolescents. The findings support the impact of even brief evidence-based interventions targeting family-level improvements for families with both TD adolescent siblings and individuals with IDD. The findings also accentuate the significance of involving both mothers and fathers in family intervention research, suggesting that different caregivers may experience both shared and unique benefits from participating.

## 1. Introduction

Adolescence is a critical developmental stage in which youths experience various changes as they try to better understand themselves and sculpt who they are as an individual [1]. The individuation of adolescents is developmentally normative; however, the transition process affects not only youths themselves but also their families [1,2]. As part of the individuation process, adolescents strive for more autonomy and independence from their parents as well as broader social relationships outside of the home [1]. Naturally, families with adolescents experience increased disagreements and conflict during this time as parents and adolescents face shifts in their dynamics and attempt to re-negotiate their boundaries [2,3,4,5,6].

Despite the increased challenges, the family environment continues to play a key role in facilitating positive adolescent development by allowing adolescents to learn and practice healthy attitudes and skills for interpersonal conflict [3,5]. It is critical that families with adolescents practice constructive conflict management and communication to be able to effectively navigate the changes and challenges throughout adolescence, facilitating better youth adjustment in turn [1,2,7].

### 1.1. Families of Individuals with Intellectual and/or Developmental Disability

Parents and siblings of individuals with intellectual and/or developmental disabilities (IDD) often encounter different hardships in their family relationships and home environment compared to families without a member with IDD [8,9,10]. To accommodate for the necessary at-home and professional care for a member with IDD, parents often experience intense caregiving burdens, elevated stress, and financial hardships [8,11,12,13]. It is important to note that the caregiving burden and related stressors fall on not only parents but also on typically developing (TD) siblings, even when the TD siblings are youths themselves. Recent reviews echo that the existing literature has consistently shown that TD youth siblings considerably partake in daily caregiving, perform more responsibilities around the house, experience unwanted pressure and worries surrounding the care for their siblings with IDD, and have limited access to quality time with parents and for themselves (e.g., doing schoolwork, meeting friends) [8,14,15,16,17,18].

### 1.2. Adjustment Problems in TD Adolescents

While these hardships affect all family members of individuals with IDD, we must highlight that TD adolescent siblings may particularly struggle as they juggle common challenges during adolescence on top of the existing responsibilities related to caring for their siblings with IDD. In turn, these struggles may affect their adjustment, resulting in internalizing and externalizing problems.

Several studies have shown that TD youth siblings of individuals with IDD are at increased risk for emotional and behavioral problems. Meyer, Ingersoll, and Hambrick [18] analyzed TD youth siblings (ages 6 to 18; *M* = 10.5; *SD* = 3.6) of children with autism spectrum disorder (ASD) and found that mother-reported internalizing and externalizing problems in TD youths were positively associated with the severity of their siblings’ ASD symptoms. Other studies that specifically focused on TD adolescent siblings also presented similar findings. Tomeny et al. [19] examined TD adolescent siblings (ages 11 to 17; *M* = 13.2) of children with ASD and found that both parent-reported (98% mothers) and self-reported levels of emotional and behavioral problems in TD adolescents were positively associated with the severity of their siblings’ ASD symptoms. Smith et al. [20] also analyzed problem behaviors in TD adolescent siblings (ages 11 to 18; *M* = 13.7) of children with ASD and found that self-reported problem behaviors were positively associated with the severity of their siblings’ ASD symptoms. Additionally, such an adverse effect on TD youth sibling adjustment could last over time. In a study on TD youth siblings (T1 age *M* = 10.3; *SD* = 4.3) of children with various IDD, Hastings [21] found that the level of problem behaviors in children with IDD at T1 significantly predicted mother-reported internalizing and externalizing problems in TD siblings two years later (T2 age *M* = 11.7; *SD* = 3.9).

### 1.3. Problems in Family Functioning Between Parents and TD Adolescents

Moreover, it is undeniable that the quality of family functioning is compromised as the family arranges their routines, time, and resources to prioritize care for the member with IDD [8,22]. Parents of individuals with IDD are likely to experience increased inter-adult conflict as they continually discuss and decide the daily and long-term care for their child [23]. Although disagreements are common in romantic relationships and not always destructive [24], interparental discord may be more stressful on parents of children with IDD because they have greater caregiving burden and elevated stress than parents of typically developing children. In 2012, Gau et al. [25] compared 151 mothers and fathers of children with ASD to 113 mothers and fathers of TD children on the levels of dyadic parental agreement, marital satisfaction, and family cohesion. The authors found that both mothers and fathers of children with ASD reported significantly lower levels of dyadic agreement compared to the parents of TD children. They also found that mothers of children with ASD reported significantly lower levels of marital satisfaction and family cohesion compared to mothers of TD children but not fathers. Although there is limited research focused on interparental conflict specific to parents of children with IDD, broader research on parents of children with other chronic illnesses show that these parents tend to experience greater problems in marital adjustment, including marital satisfaction, communication, and problem solving [26,27,28].

Furthermore, parents and TD adolescent siblings may also experience increased conflict and relationship problems. There is a possibility of spill-over effects when parental stress from marital conflict and caring for a child with IDD affects how parents behave toward TD siblings [23,29]. Then, considering how commonly TD youth siblings partake in caregiving and other responsibilities to help their parents, TD adolescents and parents are likely to engage in frequent discussion and decision making similar to that between parents. However, as part of the individuation during adolescence, TD youths may start questioning and disagreeing with the given roles and tasks assigned by their parents, resulting in increased conflict between parents and TD adolescents [17].

Although there is limited empirical study to date that has directly examined conflict processes between parents and TD adolescent siblings of individuals with IDD, there is evidence of elevated risks in their relationships. Recent reviews explained that TD adolescent siblings often reported receiving less parental support, unfair responsibilities, and less quality family time due to prioritizing the needs of their siblings with IDD [8,16,17]. In fact, parents also acknowledge the disadvantages of their TD youth. Mulroy and colleagues analyzed 327 parents of children with Down Syndrome or Rett Syndrome and found that 75% of parents reported that their TD youths indeed faced disadvantages for having a sibling with IDD, such as less attention from parents and lack of family activities appropriate for their developmental level [29].

In conclusion, parents and TD adolescent siblings are commonly burdened with caregiving responsibilities and other related stress surrounding care for their family members with IDD. In turn, parents and TD adolescent siblings are at increased risk for conflict in marital and parent–adolescent relationships as well as adolescent maladjustment. Therefore, parents and TD adolescent siblings may greatly benefit from an evidence-based intervention program targeting family conflict that promotes constructive communication between parents and TD adolescents [10,30,31].

### 1.4. Conflict Intervention for Parents and TD Adolescents

Previous intervention studies for families with IDD have primarily targeted parental benefits, including parental stress management (e.g., breathing exercises, peer support by meeting other parents of children with IDD), managing behaviors of children with IDD, or therapeutic clinical practices [30,32]. Some intervention studies that involved TD siblings focused on how to better handle stress from managing their siblings with IDD (e.g., psychoeducation about IDD symptoms, peer-group activities to better cope with and validate their circumstances of having siblings with IDD), rather than actively targeting family-wide improvements [31,33].

Additionally, there are two critical methodological limitations that persist across a large body of existing translational literature for families of individuals with IDD. First, most research depended on mother participation and survey reports; thus, we lack the understanding of whether and how fathers benefit from family intervention [30,32]. Second, previous intervention studies involving TD siblings often failed to include a control group, critically limiting their statistical validity to test true treatment effects [33]. The current study aims to address these limitations by testing a brief family conflict intervention including (a) both mothers and fathers and both TD adolescent and IDD siblings, and (b) an active control condition.

### 1.5. Current Study

Thus, this study addresses the major limitations in existing family intervention research on parents and TD adolescent siblings of individuals with IDD by testing the mothers’, fathers’, and TD adolescents’ conflict knowledge and adolescent adjustment following a brief family intervention. We analyzed 141 families of individuals with IDD and TD adolescent siblings that participated in the Supporting Parent–Adolescent Relationships and Communication (SPARC) project, a 4-week evidence-based intervention program that educated participating families on the impact of family conflict and constructive communication strategies [34]. In particular, we tested the post-test intervention effects on the mothers’ and fathers’ knowledge of marital conflict, the TD adolescents’ knowledge of family-wide conflict, and internalizing and externalizing problems in TD adolescents. Previous intervention studies that examined a precedent intervention program (Family Communication Project; FCP) for the SPARC project revealed significant short-term and long-term intervention effects on families’ knowledge of conflict [35,36] and adolescents’ internalizing and externalizing problems [35,37]. However, the findings from the FCP studies were based on community samples with only TD adolescents. The present SPARC project maintained the core conceptual and intervention components of FCP’s successful program, while further emphasizing the messages and strategies that were directly applicable to the challenges for families with children with IDD.

For the SPARC project, all family members (mothers, fathers, and TD adolescents) participated in weekly intervention sessions together. Families were randomly assigned to either a treatment condition or an active control condition. Although we anticipated that all participating families could benefit, considering the nature of active control conditions, we primarily hypothesized enhanced benefits for families in the treatment condition [35]. Therefore, we anticipated to find significant post-test intervention effects on both mothers’ and fathers’ knowledge of marital conflict, youth’s knowledge of family-wide conflict, and all family members’ reports on adjustment problems in TD adolescents. To test the hypothesized intervention effects, we performed (1) analysis of covariance (ANCOVA) and (2) gain-score analysis of variance (ANOVA). First, ANCOVA examined if the treatment and control groups significantly differed on their post-test scores of target constructs while controlling for their pre-test scores. For ANCOVA, we hypothesized that mothers, fathers, and TD adolescents in the treatment group would report greater knowledge of marital and family-wide conflict (Hypothesis 1a) and fewer adjustment problems in TD adolescents (Hypothesis 2a) compared to family members in the control group. Then, gain-score ANOVA examined if the treatment and control groups significantly differed in the level of improvement between pre-test and post-test scores. For gain-score ANOVA, we hypothesized that mothers, fathers, and TD adolescents in the treatment group would show a significantly greater level of improvement between pre-test and post-test scores on conflict knowledge (Hypothesis 1b) and TD adolescents’ adjustment (Hypothesis 2b), compared to family members in the control group. For all analyses, we examined mother, father, and adolescent reports independently to compare whether and how mothers, fathers, and adolescents differed in their intervention experiences.

## 2. Materials and Methods

### 2.1. Participants

We analyzed a community sample of 141 families that participated in a 4-week intervention program, the SPARC project. The eligibility criteria were that the participating family must (1) have a TD adolescent between the ages of 10 and 19 at the time of their pre-test assessment, and (2) a child of any age with an intellectual and/or developmental disability living in the same home with their parents and TD sibling. Additionally, parents were required to be married or living together at the time of participation and all participating family members were required to have been living together for at least three years by the time of study recruitment. Families were required to be English literate. Most parents were married (96.4%) and were biological mothers (89.3%) and biological fathers (87.6%) of the participating adolescents. The other parental relationships included stepparents, adoptive parents, foster parents, or live-in partners. Mothers were between the ages of 29 and 59 (*M* = 43.2; *SD* = 6.1) and fathers were between the ages of 28 to 62 (*M* = 44.6; *SD* = 6.6). Most parents completed either high school (mothers = 24.3%; fathers = 33.3%) or received a college degree or higher (mothers = 75.7%; fathers = 63.6%). About half of the mothers (44.3%) and most of the fathers (87.6%) worked full time. The median household income was between $75,000 and $99,999. Adolescents were between the ages of 10 and 18 (*M* = 13.0; *SD* = 2.3) and had an even gender distribution (51.4% girls). The families were predominantly White (mothers = 83.6%; fathers = 86.0%; youths = 78.6%), followed by multi-racial (mothers = 8.6%; fathers = 6.2%; youths = 14.3%). Siblings with IDD included various diagnoses: 50.4% had ASD, 16.3% had Down Syndrome, and 12.6% had an intellectual disability. About half of the siblings with IDD (53.3%) reported comorbidity with other conditions, such as ADHD (22.2%) and anxiety (10.4%). The ages of siblings with IDD ranged from 1 to 27 (*M* = 11.4; *SD* = 10.6) and 32.6% were females.

The families were recruited primarily through in-person events and posted flyers in South Bend and Fort Wayne, Indiana, and through national email and social media campaigns. Recruitment materials described the program goal of improving family communication skills as well as the eligibility criteria and compensation details. Families were eligible to receive up to $370 for completing the program and follow-up assessments and were entered into a raffle for a chance to win an iPad. The procedures and all materials associated with the SPARC project were approved by the institutional review board at the University of Notre Dame (IRB protocol ID: 17-08-4011).

### 2.2. Procedures

Families were randomly assigned to either a treatment condition (*n* = 72) or a control condition (*n* = 69) before they participated in the program. Regardless of the intervention conditions, families completed the same list of assessment questionnaires and problem-solving discussion tasks during pre-test (T1) and post-test (T2) study sessions. Although the current study focused on pre-post assessments, the families also completed similar follow-up assessments at 6 and 12 months post-intervention. During each study session, mothers, fathers, and TD adolescents independently completed self-report questionnaires. Families also completed a quadratic problem-solving discussion task involving parents, TD adolescents, and IDD siblings, followed by a dyadic problem-solving discussion task between parents without their children present.

The intervention program was designed to help families improve their knowledge of family conflict and promote more constructive communication behaviors and conflict resolution [24,34]. Accordingly, based on the previous research that examined similar intervention programs and their benefits [35,37,38], the SPARC project was expected to support adjustment and well-being for parents and TD siblings and indirectly support the treatment progress of children with IDD by strengthening the broader family system.

Families in the treatment condition received four weekly psychoeducation sessions and two communication coaching sessions between T1 and T2. Each session lasted approximately two hours. The psychoeducation sessions were interactive and covered research-based information regarding conflict, communication, family stress and security, and parent–child communication, and they introduced a communication technique called the iPad strategy. In the first three sessions, parents and TD adolescents received separate, developmentally appropriate presentations that covered similar themes. The fourth session included the children with IDD as well as the parents and TD siblings and emphasized key points from previous sessions and encouraged families to work together toward productive communication and conflict resolution. Communication coaching sessions provided individualized feedback from a family coach for each family, providing an opportunity to review and practice hands-on application of the communication techniques during psychoeducation.

Families in the control condition received a several-page newsletter that summarized the research-based information presented in the psychoeducation sessions, excluding coverage of the iPad technique. Along with the newsletter, families in the control condition received a brief, interactive overview of the program and a rubric to guide their review of the newsletter and direct their attention to specific pages pertinent to each week of the intervention program. This self-guided information was paired with weekly check-ins from a family coach conducted by phone, email, or text message, based on each family’s preference.

The SPARC project was administered by graduate students, undergraduate research assistants, and research staff trained on the manualized program. This administrative approach of the SPARC project increased program accessibility by developing a manualized intervention that did not require professional program administrators.

#### In-Person to Online Study Administration

At the beginning of the study, the SPARC project administered all of the intervention sessions and study visits in person at a research center. However, in 2020, the SPARC project fully transitioned from in-person administration to online administration due to the COVID-19 pandemic. To maintain the core components of the intervention and avoid restrictions associated with lockdowns and other COVID-related precautions, our research team developed asynchronous psychoeducation modules administered via Qualtrics. This online module replicated the content, activities, discussions, and themes that were offered in the in-person administration of the program. Our team could also track each family’s participation progress on the online platform, including details about when the modules were completed and how long it took. Importantly, the online platform continued to offer the interactive components of the psychoeducational sessions by allowing the families to submit questions, comments, and reflections on the content in real time as they reviewed it. Communication coaching sessions, which had occurred in private rooms at a community-based research center, were converted to live, recorded Zoom meetings that maintained all of the components of the in-person sessions, with the added benefit of enhanced ecological validity as families participated from their own homes. Similarly, pre- and post-test and follow-up assessment procedures were adapted to be completed via live Zoom sessions. All study sessions were recorded by a centralized recording team on Secure Zoom meetings to maintain data security and eliminate the need for physical transfer of identifiable data.

The transition during the COVID-19 pandemic to the virtual application of the program was very successful. In fact, there were unanticipated benefits of the virtual format. Online participation minimized the participating families’ challenges of arranging childcare to travel to the research site as well as parents’ concerns about environmental exposures to restricted items for their child with IDD (e.g., food dyes and ingredients, fluorescent lighting, etc.). Moreover, study visit attendance increased due to the ease of online participation, which eliminated the need for families to travel to a research center. Additionally, the online format also increased the eligible population of participants by eliminating geographic barriers to study participation. Based on these benefits, and given the painstaking efforts to maintain all of the key components of the intervention across in-person and virtual formats, the remainder of the study was conducted using the online format, even after the eventual ease of COVID-related restrictions. The subsequent analyses on group differences between the in-person and online samples revealed no significant group differences in demographic variables, key study variables, or attrition, with one exception: the median household income for the in-person sample was between $75,000–$99,999, while the median household income for the online sample was between $100,000–$124,999 (Appendix A).

### 2.3. Measures

#### 2.3.1. Intervention Conditions

The participating families were randomly assigned to either the treatment or control condition prior to study participation. Mothers, fathers, TD adolescents, and siblings with IDD were required to complete the study visit together. Adults in the treatment condition were asked to interact together in psychoeducation sessions. TD adolescents received a separate psychoeducation session that offered parallel content to the adult versions in a developmentally appropriate level for youth. Between psychoeducation sessions, parents and their TD adolescents participated in communication coaching sessions where a trained coach facilitated a conflict discussion using communication strategies that were introduced during the psychoeducation. The communicative strategy, called the iPad technique, was designed to help families slow down the conversation and move toward a mutual resolution. Parents in the control condition were asked to review the newsletter content and were encouraged to share it with their families; however, TD adolescents in the control condition did not receive tailored intervention content. Prior to the final analysis, we confirmed that the random assignment achieved no significant group differences between the families in the treatment and control conditions in their demographic and study variables at T1 (Appendix B).

#### 2.3.2. Parents’ Knowledge of Marital Conflict

Parent’s knowledge of family conflict was measured using the 19-item Parent Knowledge of Marital Conflict (PKMC) [38] questionnaire analyzing their understanding of how various aspects of marital conflict impact family members. Example items are “Disagreements are normal and common in happy marriages” and “The degree of marital conflict present in a family has a major impact on a child’s healthy development”. Mothers and fathers each completed the questionnaire about their knowledge, rating each item on a 4-point Likert scale ranging from “not at all true” (1) to “completely true” (4). The rating scales were reversed for certain items so that higher ratings would indicate better knowledge of conflict and its impact on families. Cummings and colleagues [38] demonstrated moderate to good reliability for PKMC (*α* = 0.64–0.77) with their sample of couples with young children. Additionally, Devonshire et al. [36] replicated this reliability with a sample of parents of TD adolescents that participated in the FCP (*α* = 0.64–0.72). The current study sample also demonstrated reasonable internal consistency for mothers (T1 *α* = 0.60; T2 *α* = 0.70) and fathers (T1 *α* = 0.66; T2 *α* = 0.74), comparable to the previous literature. For this study, three items were removed to strengthen the internal consistency, thus 16 items were used in the final analysis. Each parent’s score was computed using an average of 16 items.

#### 2.3.3. Adolescents’ Knowledge of Family Conflict

TD adolescents’ knowledge of family conflict was measured using the 13-item Adolescent Knowledge of Conflict Questionnaire (AKCQ). AKCQ is a parallel measure of PKMC for youth, analyzing adolescents’ understanding of how family conflict impacts family members. Example items are “Disagreements are normal and common in close relationships” and “Relationships are affected by what people do and say during disagreements.” Adolescents completed the questionnaire using a 4-point Likert scale ranging from “not at all true” (1) to “completely true” (4). The rating scales were reversed for certain items so that higher ratings would indicate better knowledge of conflict and its impact on families. Moderate internal consistency was found (T1 *α* = 0.63; T2 *α* = 0.68). For this study, three items were removed to improve the internal consistency, thus 10 items were used in the final analysis. Each adolescent’s score was computed using an average of 10 items.

#### 2.3.4. TD Adolescents’ Internalizing and Externalizing Problems

TD adolescents’ internalizing and externalizing problems were measured using the 25-item Strengths and Difficulties Questionnaire (SDQ) [39], which comprehensively examines emotional, behavioral, and social problems. The parent version and youth version administer the same 25 items; however, some items in the youth version are rephrased to be age appropriate. Example items in the parent version are “My typically developing child is generally well behaved, usually does what adults request” and “My typically developing child often lies and cheats.” Example items in the youth version are “I usually do as I am told” and “I am often accused of lying or cheating.” Parents and adolescents independently completed the questionnaire, rating each item on a 3-point Likert scale of “not true” (0), “somewhat true” (1), and “certainly true” (2). Goodman established the good reliability of the SDQ for both parent reports (*α* = 0.82–0.88) [39,40] and youth reports (*α* = 0.80) [40]. Good internal consistency was found for mothers (T1 *α* = 0.87; T2 *α* = 0.88), fathers (T1 *α* = 0.85; T2 *α* = 0.89), and TD adolescents (T1 *α* = 0.84; T2 *α* = 0.87). Each family member’s score was computed using an average of 25 items, where higher scores indicated more adjustment problems.

## 3. Results

### 3.1. Attrition

Out of the 141 families at the T1 assessment, 91 families (64.5%) had complete data pertinent to the current analysis at both the T1 and T2 assessments (T2 treatment *n* = 40; T2 control *n* = 51). For the data analyzed in the current study, the retention rates were 55.5% for the treatment group (40 out of 72 families) and 72.8% for the control group (51 out of 69 families). The retention rates were 75% for in-person participation (30 out of 40 families) and 60% for online participation (61 out of 101 families). Using ANOVA, we confirmed that the retained participants and lost participants did not significantly differ in family demographics and study variables at T1, except for the mother’s employment status. This did not pose any considerable threat to the final analysis since the mother’s employment status was not a significant covariate in any of the analysis models (Appendix C).

### 3.2. Preliminary Analyses

Prior to conducting the main analysis, we confirmed that all study variables satisfied the statistical assumptions for ANCOVA: (1) independence of covariate (i.e., pre-test score) and independent variable (i.e., intervention conditions), and (2) homogeneity of regression slopes. Additionally, preliminary analysis revealed that the demographic covariates of adolescent gender, race, and age were not significant factors explaining the variance in post-test communication quality. As a result, these demographic variables were not included in the final analysis.

### 3.3. ANCOVA Results

#### 3.3.1. Parents’ Knowledge of Marital Conflict

We found a significant intervention effect on mother-reported (*F*(1, 83) = 14.96, *p* < 0.001) and father-reported knowledge of marital conflict (*F*(1, 81) = 11.19, *p* = 0.001). Specifically, mothers in the treatment group (*M* = 3.64; *SD* = 0.03) reported significantly better knowledge of marital conflict and its impact on families compared to mothers in the control group (*M* = 3.47; *SD* = 0.03). Similarly, fathers in the treatment group (*M* = 3.45; *SD* = 0.04) reported significantly better knowledge of marital conflict and its impact on families compared to fathers in the control group (*M* = 3.27; *SD* = 0.04). The result indicated a large effect size for mother reports (partial *η*^2^ = 0.15) and a moderate effect size for father reports (partial *η*^2^ = 0.12). See Table 1.

#### 3.3.2. TD Adolescents’ Knowledge of Family Conflict

We also found a significant intervention effect on TD adolescents’ knowledge of family conflict (*F*(1, 74) = 7.04, *p* = 0.01). TD adolescents in the treatment group (*M* = 3.39; *SD* = 0.06) reported significantly better knowledge of family-wide conflict and its impact on families compared to those in the control group (*M* = 3.18; *SD* = 0.05). The effect size was medium (partial *η^2^* = 0.09). See Table 1.

#### 3.3.3. TD Adolescent’s Internalizing and Externalizing Problems

We found a significant intervention effect on father-reported TD adolescents’ adjustment problems (*F*(1, 80) = 4.28, *p* = 0.042). Fathers in the treatment group (*M* = 0.50; *SD* = 0.04) reported significantly less internalizing and externalizing problems in their TD adolescents compared to fathers in the control group (*M* = 0.62; *SD* = 0.04). The effect size was small (partial *η*^2^ = 0.05). See Table 1.

However, we did not find significant intervention effects for mother-reported and adolescent-reported adjustment problems.

### 3.4. Gain-Score ANOVA Results

#### 3.4.1. Parents’ Knowledge of Marital Conflict

The level of improvement in parents’ knowledge of marital conflict was significantly greater for the treatment group for both mothers (*F*(1, 84) = 13.89, *p* < 0.001) and fathers (*F*(1, 82) = 9.12, *p* = 0.003). For mothers, the treatment group showed an average 0.20 point increase (*SD* = 0.24) in their knowledge score between T1 and T2, while the control group showed a 0.02 point increase (*SD* = 0.20). For fathers, the treatment group showed an average 0.22 point increase (*SD* = 0.26) in their knowledge score between T1 and T2, while the control group showed a 0.03 point increase (*SD* = 0.28). The results indicated a large effect size for mother reports (Cohen’s *d* = 1.06) and a moderate effect size for father reports (Cohen’s *d* = 0.67). See Table 2.

#### 3.4.2. TD Adolescents’ Knowledge of Family Conflict

The level of improvement in TD adolescents’ knowledge of family-wide conflict was significantly greater for the treatment group (*F*(1, 75) = 6.82, *p* = 0.011). Specifically, TD adolescents in the treatment group exhibited an average 0.27 point increase (*SD* = 0.32) in their knowledge score between T1 and T2, while those in the control group had an average 0.05 point increase (*SD* = 0.41) in their knowledge score. The effect size was medium (Cohen’s *d* = 0.61). See Table 2.

#### 3.4.3. TD Adolescents’ Internalizing and Externalizing Problems

The level of improvement in father-reported TD adolescents’ adjustment problems was significantly greater for the treatment group (*F*(1, 81) = 4.25, *p* = 0.042). Fathers in the treatment group reported an average 0.03 point decrease (*SD* = 0.23) in their TD adolescents’ internalizing and externalizing problems, while fathers in the control group reported an average 0.09 point increase (*SD* = 0.30) in TD adolescents’ adjustment problems. The effect size was small (Cohen’s *d* = 0.45). See Table 2.

However, there were no significant findings for mother and adolescent reports.

## 4. Discussion

The current study analyzed whether a brief family conflict intervention could improve parents’ and TD adolescent siblings’ knowledge of family conflict as well as TD adolescents’ adjustment problems. This is the first study to examine an intervention focused on improving marital and family-wide conflict and communication for parents and TD adolescent siblings of individuals with IDD. Importantly, this study examined mother, father, and adolescent reports independently to understand whether and how family members experience intervention benefits differently.

Our findings fully supported Hypotheses 1a and 1b; however, they only partially supported Hypotheses 2a and 2b. As expected, for Hypothesis 1a, mothers, fathers, and TD adolescents in the treatment condition reported significantly better knowledge of martial and family-wide conflict at T2 compared to those in the control condition, while controlling for their T1 reports. Moreover, for Hypothesis 1b, mothers, fathers, and TD adolescents in the treatment condition showed greater levels of improvement in conflict knowledge between pre-test and post-test scores compared to those in the control condition. For Hypothesis 2a, fathers in the treatment condition reported significantly less internalizing and externalizing problems in their TD adolescents at T2 compared to fathers in the control condition, while controlling for their T1 reports. Similarly, for Hypothesis 2b, fathers in the treatment condition reported a significantly greater level of Improvement in TD adolescents’ adjustment problems compared to fathers in the control condition. However, mothers and TD adolescents in the treatment and control conditions did not significantly differ in either their post-test mean scores of adjustment problems in TD adolescents or the level of change between pre-test and post-test reports.

### 4.1. Implications

First, the current findings of significant intervention effects on improving families’ knowledge of marital and family-wide conflict are consistent with previous studies that analyzed the precedent program of the SPARC project with a typically developing family sample. In alignment with our findings for Hypothesis 1a, Bergman et al. [35] found that mothers and fathers in treatment conditions scored significantly higher on their knowledge of marital conflict than parents in control conditions at post-test assessments. Additionally, similar to our findings for Hypothesis 1b, Devonshire et al. [36] found that mothers and fathers in treatment conditions exhibited greater improvement in their knowledge of marital conflict between pre-test and post-test assessments compared to those in control conditions. This series of empirical findings evidence that a brief family intervention over four weeks can effectively improve a family’s understanding of their conflict and its impact on their well-being. Furthermore, the current findings support that the intervention benefits found for typically developing families in the previous studies could be generalized to at-risk families with greater caregiving stress and responsibilities.

Next, we found that fathers, but not mothers, in the treatment condition reported a significant reduction in TD adolescent siblings’ adjustment problems after participating in the four-week intervention. Previous intervention studies that examined both mothers and fathers may provide possible explanations for such differential effects between parents. First, Hoegler et al. [41] found that the FCP intervention resulted in significant improvements in father–adolescent attachment but not in mother–adolescent attachment. The authors explained that fathers were expected to benefit more from participating in the intervention based on the fathering vulnerability hypothesis, which explains that fathers are more susceptible to negative consequences of family conflict [41]. Next, Yang et al. [42] revealed that fathers and mothers reported different improvements in communicating with their adolescents following FCP completion, where fathers reported reduced problems in communication, while mothers reported increased openness in communication. In this study, the authors delineated that the different findings between parents may be due to the different relationships that adolescents tend to have with their mothers versus fathers, such as frequency of interaction and emotional closeness. Therefore, differential treatment effects for mothers and fathers may be expected in family intervention research.

Moreover, past survey studies that examined the relationship between family functioning and TD youth siblings’ adjustments may corroborate why fathers in the current study reported reduced adjustment problems in TD siblings. In a survey study, Long et al. [43] found that open and expressive communication between parents and TD youth siblings of individuals with IDD predicted less internalizing problems and better adjustment as self-reported by TD youth siblings (age *M* = 12.1; *SD* = 2.5). Similarly, Giallo and Gavidia-Payne [44] tested that the level of family problem solving was negatively correlated with and predicted by parent-reported (90% mothers) TD youth siblings’ adjustment problems (age *M* = 11.6; *SD* = 2.7). The authors also found that family problem solving was a significant mediator for the negative relationship between socioeconomic status and TD youth siblings’ adjustment problems. Hence, the SPARC project’s program content focused on improving family conflict communication and resolution may have resulted in better adolescent adjustment noticed by fathers, as shown in previous research.

Lastly, we consider the question surrounding the significant intervention effect found for TD adolescents’ adjustment specifically from father reports but not from mother or adolescent reports. This pattern contradicts the past findings based on mother [37,44] and youth self-reports [43]. However, the present study breaks new ground by including father reports, which were not included in past studies. Scholars have continually emphasized that the lack of research focus on fathers, including their study participation, is a persistent limitation in the literature surrounding families of individuals with IDD [8,30,32]. Although there are no comparable studies to dissect our father-specific findings for a similar sample, our recent study based on multi-informant analysis of the FCP intervention effect might help to illustrate the basis for the variations in results across different family members. Yang et al. [42] compared mothers, fathers, and adolescents on the FCP’s intervention effect for parent–adolescent communication quality and found that mothers and adolescents reported intervention effects for only the positive quality (i.e., openness) while fathers reported intervention effects for only the negative quality (i.e., problems). The authors discussed that the different findings across family members may be due to the underlying differences in mother–adolescent and father–adolescent dynamics. The authors explained that the improvement in openness may have been more easily achieved for mother–adolescent dyads because mothers already tend to be more open and expressive with their children compared to fathers. Moreover, fathers may have reported exclusive intervention benefits for communication problems because they are less familiar with practicing open communication with their teens compared to mother–adolescent dyads, thus considering the reduction in communication problems to be more considerable than did mothers and adolescents. The previous findings and discussion of the inherent differences between mother–adolescent and father–adolescent communication quality may explain why the current study only found significant reductions in TD adolescents’ adjustment problems in father reports but not in mother or youth reports.

### 4.2. Strengths, Limitations, and Future Direction

This study tackled critical gaps and limitations in the existing intervention research on parents and TD adolescent siblings of individuals with IDD. First, we reviewed evidence that parents and TD adolescent siblings are at increased risk of problems in family functioning and adolescent adjustment due to the intense caregiving burden and related stressors [8,11,13,14,16,17,31]; however, previous intervention studies focused on programs targeting parent-specific (e.g., breathing exercise for stress management) or sibling-specific (e.g., psychoeducation about IDD) factors, overlooking the importance of family-wide changes. We tackled this critical gap in the literature by evaluating a family intervention program targeting family conflict and communication. Our findings showed that a brief conflict intervention over four weeks significantly improved families’ knowledge of marital and family-wide conflict as well as father-reported adjustment problems in TD adolescent siblings. We emphasize that more family intervention research should involve both parents and TD siblings, rather than focusing on a single family member.

Next, this study examined both mothers and fathers, as opposed to most of the previous intervention research that depended on mothers for study participation and program evaluation [31,33]. Very limited previous research that recruited either mothers or fathers as parents still had over 90% of parents being mothers [44]. By contrast, our sample recruited both mothers and fathers to participate in the study together. Our multi-informant findings highlight that while mothers and fathers may benefit similarly from participating in the same program (i.e., improved knowledge of marital conflict), they may also experience different benefits (i.e., only fathers reported significant reduction in TD adolescents’ adjustment problems). Additionally, the shared improvements of mothers and fathers in knowledge of marital conflict would not be redundant but rather synergetic for further benefits for families beyond the immediate intervention effect seen in this study. This analytical decision tackled the gap in family intervention literature of lacking an understanding of the father’s study experiences. Altogether, our study strongly corroborates the significance of intervention studies to include both mothers and fathers and to encourage both-parent participation for applicable households. We recommend future studies to quantitatively and qualitatively examine how similarly or differently mothers and fathers perceive family intervention experiences. The findings will help researchers to better understand the mechanism behind the benefits of both-parent participation.

Furthermore, the current study administered both ANCOVA and gain-score ANOVA to evaluate the intervention efficacy of the SPARC project. While both analyses are commonly used in psychological research to test treatment effects, it is less common for studies to conduct both analyses despite their inferential differences. ANCOVA examined post-test group mean differences, while controlling for pre-test scores; this is a robust analytical choice for evaluating if treatment and control conditions significantly differ on their average scores after the treatment. Gain-score ANOVA examined group differences in the level of change between pre-test and post-test scores; this is an appropriate analysis for testing if the treatment condition exhibits significantly greater improvement in target scores between before and after treatment compared to the control condition, or vice versa. Therefore, the current study was able to confirm that the families in the treatment condition not only had significantly better level of knowledge and less adjustment problems after program participation but also exhibited significantly greater levels of change in those target constructs over four weeks compared to families in the control condition. The consistent empirical support from distinct statistical analyses further strengthened the evidence for the intervention efficacy of the SPARC project for parents and TD adolescent siblings of individuals with IDD [45,46].

A few limitations of this study must be addressed. First, although the sample size of 91 at T2 is statistically sufficient and larger than most of the previous intervention studies, the attrition rate was noticeable, considering the sample size at T1 was 141. There were three major challenges for participant retention: (1) a significant portion of the SPARC project was administered during the COVID-19 pandemic, (2) the SPARC project required both parents and TD adolescent siblings to attend four weekly intervention sessions and all study visits together, and (3) families of individuals with IDD must arrange childcare plans to be able to attend and participate in intervention research. Despite the inevitable attrition, we strongly believe that offering the online intervention platform was critical to retaining a moderate portion of families against these major challenges. We encourage future longitudinal studies to explore online administration options for intervention delivery and data collection, especially when working with families of individuals with IDD. Second, the current study only examined the post-test intervention effect immediately following program participation. This lacks analysis of whether the observed intervention effects are temporary or could last over time. Future research should evaluate follow-up assessments beyond post-test reports to be able to evaluate the long-term intervention efficacy. Third, this study focused on group difference analyses and did not evaluate the potential mechanisms underlying the intervention effect. In other words, our findings showed that the SPARC project could significantly improve families’ knowledge of conflict and father-reported TD adolescent sibling’s adjustment problems; however, the current analysis did not directly analyze which components of the SPARC program or which specific changes in families during participation allowed them to achieve such improvements. Specifically, future research would merit from conducting mediation or moderation analysis exploring the potential factors (e.g., change in observed conflict communication behaviors or TD adolescents’ emotional security) behind the intervention effects. Fourth, the current study had an attrition rate of 35.5%. Although the large initial sample size helped to retain 91 families for the final analysis, this inherent challenge in longitudinal research merits discussion. Future studies should implement various approaches to improve participant retention, such as regular study reminders from experimenters. Lastly, the current sample was largely socio-culturally homogeneous as the majority of the participating families were two-parent households, predominantly White, and middle to upper class. The participant homogeneity may be partially explained by being representative of major Midwestern cities that were targeted during study recruitment prior to the pandemic. Nonetheless, these sample characteristics limit the generalizability of the current findings to other family dynamics, such as single-parent households, culturally and/or ethnically diverse families, and families with socioeconomic challenges. We hope that this study informs future research to recruit more diverse family samples for stronger external validity and greater translational benefit to reach more families in our communities.

## 5. Conclusions

Adolescence often presents new challenges to both youths themselves and their parents. Even though a certain level of conflict is developmentally normative during this time, it may be debilitating for parents and TD adolescent siblings of individuals with IDD who are already experiencing burdened responsibilities and high levels of stress. An evidence-based intervention focusing on family-level factors, such as conflict and communication strategies, could greatly help these families to better understand and navigate the common challenges together, promoting positive adolescent adjustment in turn. More intervention studies should focus on family-wide benefits, rather than targeting a single family member, such as mothers. Researchers should also continue to analyze the importance of father participation in interventions for families of individuals with IDD.

## Figures and Tables

**Table 1:** ANCOVA: comparing post-test group means.

	Treatment		Control			
	*M*	*SD*	*M*	*SD*	*F*	*Partial η^2^*
*Knowledge about family conflict (Hypothesis 1a)*						
Mother-reported	3.64	0.03	3.47	0.03	*F*(1, 83) = 14.96 ***	0.15
Father-reported	3.45	0.04	3.27	0.04	*F*(1, 81) = 11.19 ***	0.12
TD adolescent-reported	3.39	0.06	3.18	0.05	*F*(1, 74) = 7.04 **	0.09
*TD adolescents internalizing and externalizing problems (Hypothesis 2a)*						
Mother-reported	0.49	0.03	0.49	0.02	*F*(1, 83) = 0.01	0.00
Father-reported	0.50	0.04	0.62	0.04	*F*(1, 80) = 4.28 *	0.05
TD adolescent-reported	0.51	0.03	0.53	0.03	*F*(1, 71) = 0.16	0.002

* *p* ≤ 0.05, ** *p* ≤ 0.01, *** *p* ≤ 0.001.

**Table 2:** Gain-score ANOVA: group comparison for level of change between pre-test and post-test.

	Treatment		Control			
	*M*	*SD*	*M*	*SD*	*F*	*Cohen’s d*
*Knowledge about family conflict (Hypothesis 1b)*						
Mother-reported	0.20	0.24	0.02	0.02	*F*(1, 84) = 13.89 ***	1.06
Father-reported	0.22	0.26	0.03	0.28	*F*(1, 82) = 9.12 **	0.67
TD adolescent-reported	0.27	0.32	0.05	0.41	*F*(1, 75) = 6.82 **	0.61
*TD adolescents internalizing and externalizing problems (Hypothesis 2b)*						
Mother-reported	−0.02	0.14	−0.02	0.17	*F*(1, 84) = 0	0.00
Father-reported	−0.03	0.23	0.09	0.30	*F*(1, 81) = 4.25 *	0.45
TD adolescent-reported	−0.05	0.20	−0.04	0.13	*F*(1, 72) = 0.08	0.06

* *p* ≤ 0.05; ** *p* ≤ 0.01; *** *p* ≤ 0.001.

## Data Availability

Access to the SPARC project data and materials may be available upon request to Dr. Cummings at ecumming@nd.edu.

## Appendix A

ANOVA group comparison analysis: In-person vs. Online administration on pre-test data.

**Table A1:** In-person vs. Online administration on family demographic at pre-test assessment.

	In-Person		Online			
	*M*	*SD*	*M*	*SD*	*F (1, 138)*	*p*
**Demographic**						
*Age*						
Mothers	42.20	6.18	43.57	6.07	1.44	0.232
Fathers ^a^	44.16	6.24	44.84	6.83	0.27	0.604
TD Adolescents	12.59	1.92	13.17	2.43	1.81	0.181
*Education level*						
Mothers	1.70	0.46	1.78	0.42	0.99	0.322
Fathers ^a^	1.59	0.55	1.61	0.55	0.02	0.896
*Employment status*						
Mothers	2.15	1.23	2.01	1.28	0.35	0.556
Fathers ^a^	1.51	1.30	1.22	0.86	2.28	0.134
*Median household income*	8.62	1.68	9.27	1.70	4.19 *	0.043
*Adolescent gender*	1.57	0.50	1.49	0.50	0.82	0.367

^a^ *F*(1, 127), * *p* < 0.05.

**Table A2:** In-person vs. Online administration on study variables at pre-test assessment.

	In-Person		Online			
	*M*	*SD*	*M*	*SD*	*F*	*p*
**Study variables**						
*Knowledge about family conflict*						
Mother-reported	3.43	0.25	3.44	0.26	*F*(1, 136) = 0.02	0.879
Father-reported	3.28	0.31	3.21	0.33	*F*(1, 126) = 1.32	0.252
TD adolescent-reported	3.06	0.40	3.12	0.44	*F*(1, 122) = 0.51	0.475
*Youth behavior problems*						
Mother-reported	0.56	0.31	0.48	0.31	*F*(1, 136) = 1.89	0.172
Father-reported	0.60	0.28	0.53	0.32	*F*(1, 126) = 1.56	0.215
TD adolescent-reported	0.58	0.24	0.58	0.29	*F*(1, 118) = 0	0.988

## Appendix B

ANOVA group comparison analysis: Treatment vs. Control on pre-test data.

**Table A3:** Treatment vs. Control on family demographic at pre-test assessment.

	Treatment		Control			
	*M*	*SD*	*M*	*SD*	*F (1, 138)*	*p*
**Demographic**						
*Age*						
Mothers	43.31	5.91	43.04	6.37	0.06	0.801
Fathers ^a^	44.30	6.43	45.03	6.87	0.40	0.531
Adolescents	12.83	2.13	13.18	2.47	0.84	0.361
*Education level*						
Mothers	1.70	0.46	1.75	0.44	0.04	0.849
Fathers ^a^	1.62	0.54	1.68	0.47	2.78	0.098
*Employment status*						
Mothers	2.11	1.42	1.99	1.09	0.34	0.559
Fathers ^a^	1.34	0.99	1.40	1.37	0.07	0.799
*Median household income*	9.04	1.67	9.13	1.77	0.10	0.751
*Adolescent gender*	1.53	0.50	1.50	0.50	0.11	0.745

^a^ *F*(1, 127).

**Table A4:** Treatment vs. Control on study variables at pre-test assessment.

	Treatment		Control			
	*M*	*SD*	*M*	*SD*	*F*	*p*
**Study variables**						
*Knowledge about family conflict*						
Mother-reported	3.41	0.26	3.47	0.25	*F*(1, 136) = 2.15	0.145
Father-reported	3.22	0.31	3.25	0.33	*F*(1, 126) = 0.19	0.667
TD adolescent-reported	3.09	0.42	3.11	0.43	*F*(1, 122) = 0.12	0.732
*Youth behavior problems*						
Mother-reported	0.51	0.31	0.50	0.32	*F*(1, 136) = 0.09	0.772
Father-reported	0.57	0.33	0.53	0.29	*F*(1, 126) = 0.43	0.515
TD adolescent-reported	0.57	0.25	0.60	0.30	*F*(1, 118) = 0.43	0.516

## Appendix C

ANOVA group comparison analysis: Families that only completed T1 vs. families that also completed T2 on pre-test data.

**Table A5:** Families that only completed T1 vs. families that also completed T2 on family demographics at pre-test assessment.

	Only T1		Completed T2			
	*M*	*SD*	*M*	*SD*	*F (1, 138)*	*p*
**Demographic**						
*Age*						
Mothers	44.28	6.00	42.57	6.13	2.55	0.112
Fathers ^a^	44.57	6.68	44.67	6.67	0.01	0.938
Adolescents	13.34	2.34	12.81	2.28	1.70	0.194
*Education level*						
Mothers	1.68	0.47	1.80	0.40	1.21	0.273
Fathers ^a^	1.53	0.60	1.64	0.53	2.53	0.114
*Employment status*						
Mothers	2.34	1.33	1.89	1.20	4.18 *	0.043
Fathers ^a^	1.35	1.10	1.28	0.98	0.13	0.721
*Family income*	8.98	1.83	9.14	1.65	0.29	0.589
*Adolescent gender*	1.54	0.50	1.50	0.50	0.20	0.653

^a^ *F*(1, 127), * *p* < 0.05.

**Table A6:** Families that only completed T1 vs. families that also completed T2 on study variables at pre-test assessment.

	Only T1		Completed T2			
	*M*	*SD*	*M*	*SD*	*F*	*p*
**Study variables**						
*Knowledge about family conflict*						
Mother-reported	3.45	0.27	3.43	0.25	*F*(1, 136) = 0.20	0.659
Father-reported	3.25	0.33	3.23	0.32	*F*(1, 126) = 0.11	0.736
TD adolescent-reported	3.05	0.49	3.13	0.39	*F*(1, 122) = 0.90	0.344
*Youth behavior problems*						
Mother-reported	0.50	0.32	0.51	0.31	*F*(1, 136) = 0.01	0.933
Father-reported	0.58	0.34	0.54	0.29	*F*(1, 126) = 0.52	0.474
TD adolescent-reported	0.62	0.26	0.57	0.28	*F*(1, 118) = 0.81	0.371
